# Development of a Novel Prognostic Signature Based on Antigen Processing and Presentation in Patients with Breast Cancer

**DOI:** 10.3389/pore.2021.600727

**Published:** 2021-04-01

**Authors:** Aoshuang Qi, Mingyi Ju, Yinfeng Liu, Jia Bi, Qian Wei, Miao He, Minjie Wei, Lin Zhao

**Affiliations:** ^1^ Department of Pharmacology, School of Pharmacy, China Medical University, Shenyang, China; ^2^ Liaoning Key Laboratory of Molecular Targeted Anti-tumor Drug Development and Evaluation China Medical University, Shenyang, China; ^3^ Department of Breast Surgery, The First Hospital of Qinhuangdao, Qinhuangdao, China

**Keywords:** antigen processing and presentation, breast cancer, cell biology, prognostic, survival

## Abstract

**Background:** Complex antigen processing and presentation processes are involved in the development and progression of breast cancer (BC). A single biomarker is unlikely to adequately reflect the complex interplay between immune cells and cancer; however, there have been few attempts to find a robust antigen processing and presentation-related signature to predict the survival outcome of BC patients with respect to tumor immunology. Therefore, we aimed to develop an accurate gene signature based on immune-related genes for prognosis prediction of BC.

**Methods:** Information on BC patients was obtained from The Cancer Genome Atlas. Gene set enrichment analysis was used to confirm the gene set related to antigen processing and presentation that contributed to BC. Cox proportional regression, multivariate Cox regression, and stratified analysis were used to identify the prognostic power of the gene signature. Differentially expressed mRNAs between high- and low-risk groups were determined by KEGG analysis.

**Results:** A three-gene signature comprising HSPA5 (heat shock protein family A member 5), PSME2 (proteasome activator subunit 2), and HLA-F (major histocompatibility complex, class I, F) was significantly associated with OS. HSPA5 and PSME2 were protective (hazard ratio (HR) < 1), and HLA-F was risky (HR > 1). Risk score, estrogen receptor (ER), progesterone receptor (PR) and PD-L1 were independent prognostic indicators. KIT and ACACB may have important roles in the mechanism by which the gene signature regulates prognosis of BC.

**Conclusion:** The proposed three-gene signature is a promising biomarker for estimating survival outcomes in BC patients.

## Introduction

Among females, breast cancer (BC) is the most commonly diagnosed cancer and the leading cause of cancer deaths (11.6%) [1]. The treatment of patients with early BC involves three main types of treatment: surgery, systemic treatment, and radiotherapy [2–4]. In clinical practice, existing clinical pathological parameters are not sufficient to inform treatment decisions for all BC patients [5].

Immunotherapy has proven to be an effective method for treating various cancers, including BC. BC is certainly immunogenic, and refined immunotherapeutic manipulations have been shown to be effective in a significant proportion of cancer patients [6–9].

Recognition of tumor antigen by specific T cells is a necessary prerequisite for the induction of effective anti-tumor immune responses. Antigen processing and presentation is the first step in the activation of the immune response and a major cellular mechanism through which cells are monitored by the immune system [10–12]. Failure to recognize antigens effectively and allowing them to develop can contribute to the development of tumors [13, 14]. Thus, understanding the presentation and processing of antigens has important implications for exerting immunologic function of naïve and memory T cells [15]. One difficulty in assessing prognosis in BC and other cancers is the complex interplay between immune cells and cancer. Thus, there are potential applications for immune-based prognostic signatures in BC. Recent transcriptomic studies on primary BC samples have shown that gene signatures related to activation of adaptive and innate immunity can have prognostic and predictive value [16, 17]. However, no signature has been identified that can predict overall survival (OS) in BC patients by systematic evaluation of genes related to antigen processing and presentation. Therefore, it is essential to develop an immune signature based on antigen processing and presentation and have prognostic ability in BC.

Research on biomarkers that can reliably estimate disease prognosis and patient survival has developed rapidly. On the one hand, several single-gene biomarkers have shown strong potential for detection and prognostic prediction in BC. For example, PTEN, PXDNL, NUF2, RRM2, BIRC5, and CDC20 can be used as predictive biomarkers for prognosis of BC [18–23]. On the other hand, multi-gene signatures have been authenticated as useful tools to predict prognosis in cancer patients [24,25]. Considering the important roles of antigen processing and presentation in the immune process and in tumor development, we aimed to develop a novel, reliable, and effective signature based on genes related to antigen processing and presentation to predict the survival and prognosis of BC patients.

## Methods

### Patient Data Sources and Workflow

We downloaded raw HTSeq count data from The Cancer Genome Atlas (TCGA; https://portal. gdc.cancer.gov/). Ensembl and DESeq were used for transcript annotation, and data were normalized using R version 3.6.3. Data were from 1090 BC patients and 113 noncancerous tissues (503 patients with complete clinical information). Supplementary Table S1 shows the general clinical characteristics of BC patients. Gene Expression Omnibus (GEO) original dataset GSE42568, containing BC gene expression profiles, was downloaded from the GEO database (https://www.ncbi.nlm.nih.gov/geo/). This dataset included 104 samples, which were used to validate our results (Supplementary Table S2).

### Construction and Confirmation of Prognostic Signature

**FIGURE 1:**
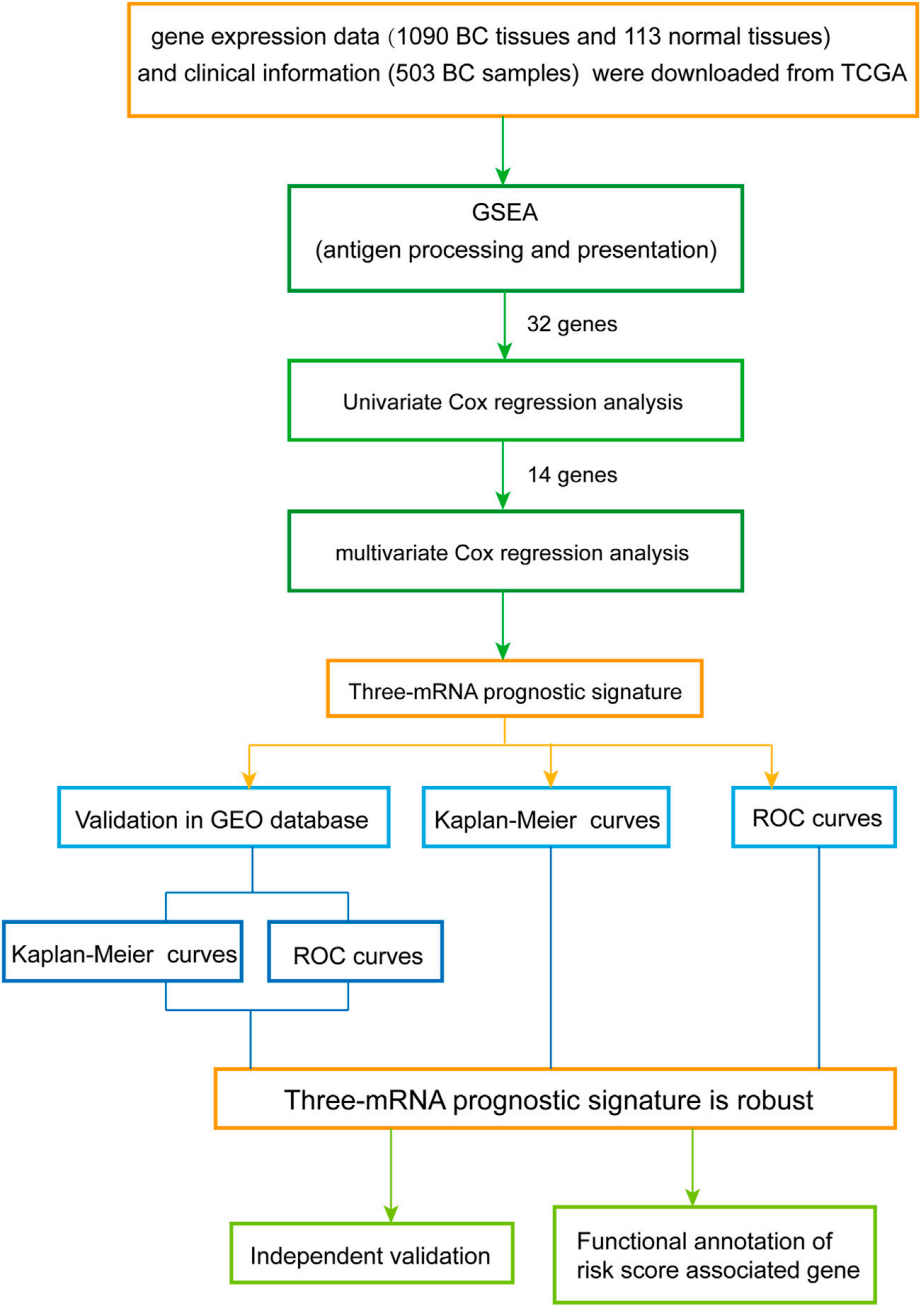
Overview of the analytic pipeline of this study.

The overall design and flow diagram of this study are presented in Figure 1. As antigen processing and presentation have a significant impact on tumor progression, gene set enrichment analysis (GSEA) (http://www.broadinstitute.org/gsea/index.jsp) was performed to explore genes enriched in antigen processing and presentation pathways that showed significant differences between noncancerous tissues and BC tissues. Subsequently, univariate Cox regression analysis was performed for the 32 genes identified by GSEA as contributing to the enrichment trends (Supplementary Table S3), resulting in 14 genes (*p* < 0.05) (Supplementary Table S4). Multivariate Cox analysis was used to further examine the links between the expression profiles of the corresponding 14 mRNAs and patients’ OS; three mRNAs (HSPA5, PSME2, HLA-F) were verified as independent BC prognostic indicators (Supplementary Table S5). Thus, a prognostic signature was constructed for BC.

Next, a risk score was calculated for each BC patient according to the expression levels of the genes (expi) and the coefficients of the multivariate Cox regression analysis (bi). The formula used was as follows:

$$\text{risk score}=\sum_{i=1}^{n}\exp i*bi.$$

Subsequently, receiver operating characteristic (ROC) curves were plotted based on the risk score and the survival status of each patient to assess the predictive accuracy of the gene signature. We constructed the ROC curves using the SPSS software, and used the maximum point of the curve (sensitivity + specificity −1) as the cutoff value for the risk score; this value was then used to divide BC patients into high- and low-risk groups (cutoff value = 1.35). Kaplan-Meier (K-M) curves were constructed to compare the OS of BC patients between the high- and low-risk groups.

### Gene Set Enrichment Analysis

GSEA was used to study whether the identified gene set (antigen processing and presentation) showed significant differences between paracancerous tissues and BC tissues. Combining the localization, nature, and function of existing genes, GSEA was used to build a database (Molecular Signatures database, MSigDB) on the basis of information such as biological significance. The antigen processing and presentation gene set is located in CP: KEGG (canonical pathways) of C2. The systematic name of this gene set is m16004. The expression levels of 34,224 mRNAs in noncancerous tissues and in BC samples were analyzed. The parameters were set as follows: number of per mutations: 1,000; collapse dataset to gene symbols: false; permutation type: gene set.

### cBioPortal Database

The cBioPortal for Cancer Genomics (http://cbioportal.org) provides a Web resource for exploring, visualizing, and analyzing multidimensional cancer genomics data. The query function was used to investigate the proportions of the three genes changing type in 6502 BC patients (including MSK, Duke-NUS, METABRIC, MSKCC, SMC, British Columbia, Broad, Sanger, TCGA, INSERM, and provisional studies).

### Timer

TIMER (https://cistrome.shinyapps.io/timer/) is a website for systematic analysis of immune infiltrates across diverse cancer types. Here, TIMER was used to investigate the links between expression of the three mRNAs and abundance of immune infiltrates.

### Processing of Cox Regression Analysis Data

Immunohistochemistry data of ER, PR, and HER2 were obtained from the clinical data downloaded from TCGA, and BC patients were divided into positive and negative groups. The expression data of PD-L1(CD274), CD4 and CD8 were obtained from the gene expression profile downloaded from TCGA. The SPSS software was used to find cutoff values for PD-L1 (1.12), CD4 (2.03) and CD8 (2.47) gene expression, and BC patients were divided into the high expression and low expression group.

### Pathway Analysis

The DAVID (Database for Annotation, Visualization and Integrated Discovery) database (https://david.ncifcrf.gov/) was used to perform functional analysis of the differentially expressed mRNAs between the high- and low-risk groups and to map the corresponding genes to Kyoto Encyclopedia of Genes and Genomes (KEGG) pathways. The results were processed using ImageGP (http://www.ehbio.com/ImageGP/index.php/Home/Index/index.html).

### Establishment of Protein–Protein Interaction Network

An interaction network of the protein products of the mRNAs that were differentially expressed between the high- and low-risk groups was established using STRING (http://string-db.org/cgi/input.pl). and the Cytoscape 3.7.0 software (Institute of Systems Biology, Seattle, WA, United States).

### Statistical Analysis

The expression profiles of 34,224 mRNAs were normalized by DESeq for further analysis. Univariate Cox regression analysis was used to calculate the associations between mRNA expression levels and patient OS. *p* < 0.05 was considered to indicate a significant association. The candidate genes were fitted using stepwise multivariate Cox proportional regression to identify the predictive model with the best explanatory and informative efficacy. Differences in patient OS between the high-risk group and the low-risk group were assessed by K-M survival analysis, with comparisons by log-rank test. All the statistical analyses were performed with SPSS16.0 and GraphPad Prism7 software. Multiple hypothesis testing correction for *p*-values was performed based on the false discovery rate.

## Results

### Detecting Prognostic Genes from the Antigen Processing and Presentation Gene Set

**FIGURE 2:**
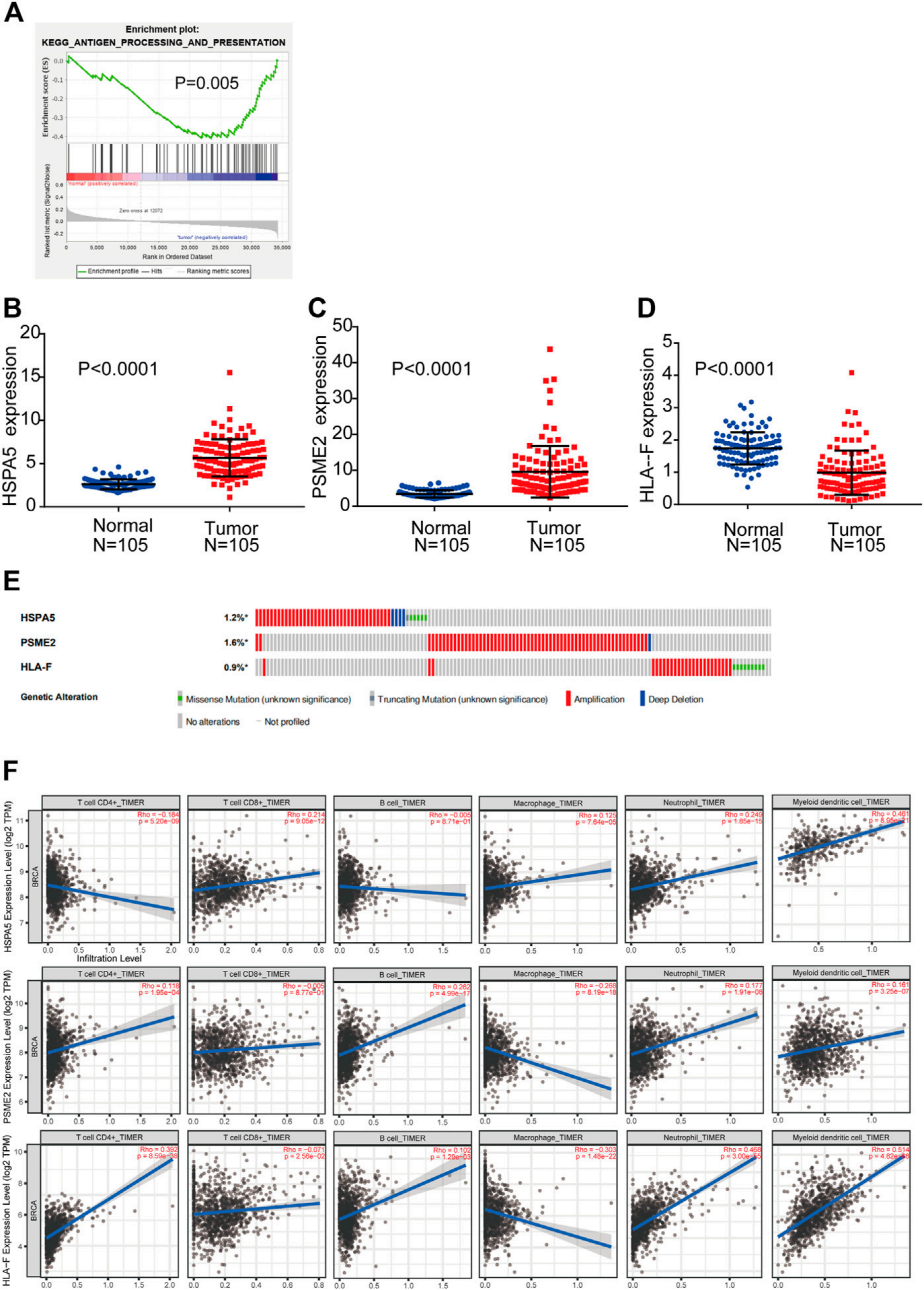
Detection of prognostic genes from the antigen processing and presentation gene set **(A)** Enrichment plots of antigen processing and presentation-related genes that were significantly differentially expressed between normal and BC tissues by GSEA **(B–D)** Expression of three genes (HSPA5, PSME2, HLA-F) using both TCGA-BRCA and matched normal breast tissue data (105 tumor samples; 105 normal samples) **(E)** Selected genes’ alterations based on 6,502 clinical BC samples **(F)** Association between abundance of immune infiltrates and three mRNAs.

GSEA was performed to explore whether the antigen processing and presentation-related gene set showed statistically significant differences between BC tissues and adjacent normal tissues. We found that the gene set was significantly enriched in BC tissues with normalized *p* = 0.005 (Figure 2A). Then, Cox regression analysis was used to verify that three mRNAs (HSPA5, PSME2, HLA-F) were independent BC prognostic indicators. The screened mRNAs were classified as either risky type (HLA-F) with HR > 1 and shorter OS, or protective type (HSPA5, PSME2) with HR < 1 and longer OS.

We compared the differential expression of the three genes in adjacent normal tissues and BC tissues (n = 105). We found that HSPA5 and PSME2 were significantly upregulated whereas HLA-F was significantly downregulated in tumor tissues compared with normal tissue (*p* < 0.0001, Figures 2B–D). We then analyzed changes in the three selected genes using BC samples from the cBioPortal database. The HSPA5 gene showed 1.2% change, including 37 examples of amplification, four examples of deep deletion, one truncating mutation, and five missense mutations. The PSME2 gene showed a 1.6% change, including 62 examples of amplification and one example of deep deletion. The HLA-F gene showed 0.9% change, including 25 examples of amplification and nine examples of missense mutation (Figure 2E).

We used TIMER to investigate the link between expression of the three mRNAs and abundance of immune infiltrates. The expression of HSPA5 and the abundance of CD4^+^ T cells showed negative linear relationship. There is no significant linear relationship between HSPA5 and B cells. The expression of HSPA5 and the abundance of other immune cells showed positive linear relationship. Besides macrophages (negative linear relationship) and CD8^+^ T cells (no significant linear relationship), the expression of PSME2 and the abundance of other immune cells showed positive linear relationship. The expression of HLA-F and the abundance of CD8^+^ T cells and macrophages showed negative linear relationship. The expression of HLA-F and the abundance of other immune cells showed positive linear relationship. These results indicate that the expression of the three mRNAs was associated with immune cell infiltration (Figure 2F).

### Three-mRNA Signature to Predicting Patients’ Outcomes

**FIGURE 3:**
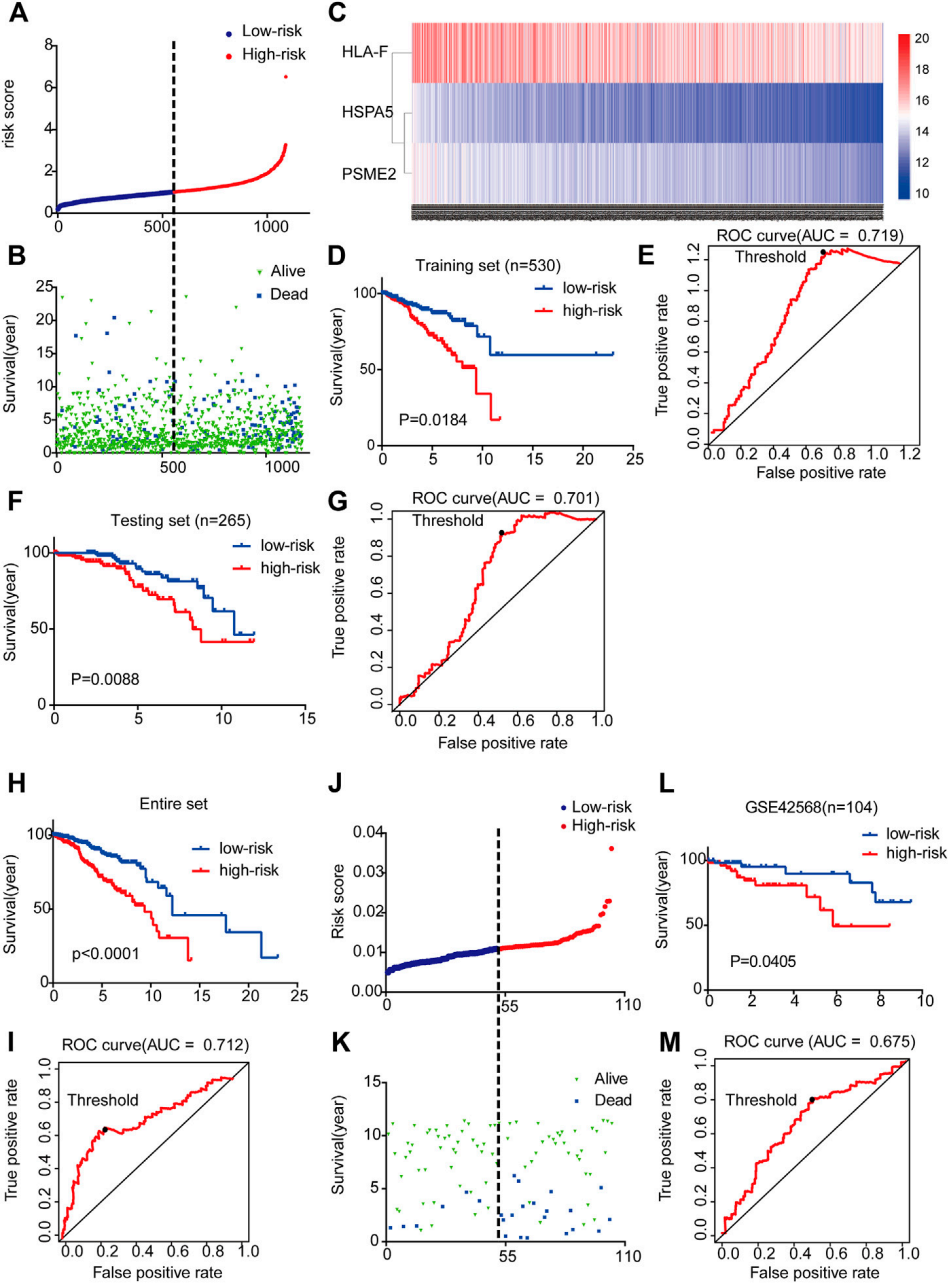
Three-mRNA signature associated with risk score predicted OS in BC patients **(A)** mRNA risk score distribution for each patient from TCGA dataset **(B)** OS time of patients in order of risk score based on TCGA **(C)** Heatmap of three selected genes’ expression profiles **(D–I)** Kaplan-Meier and ROC curves for high-risk and low-risk BC patients based on TCGA data (5-years) **(J)** mRNA risk score distribution for each patient of the GSE42568 dataset **(K)** OS time of patients in order of risk score based on GSE42568 dataset **(L-M)** Kaplan-Meier and ROC curves for high-risk and low-risk BC patients based on GSE42568 dataset (5-year).

By integrating the expression profiles of the three mRNAs and estimated regression coefficients obtained from the multivariate Cox regression analysis, we constructed a prognostic signature as follows: risk score = (−0.439 × expression value of HSPA5) + (0.213 × expression value of HLA-F) + (−0.414 × expression value of PSME2). We calculated the risk score for each patient and ranked them by risk score in increasing order (Figure 3A). The OS time (in years) of each patient is shown in Figure 3B; the mortality rates of patients with high risk scores were higher than those of patients with low risk scores. A heatmap was used to illustrate the expression profile of the three mRNAs (Figure 3C).

To examine the relationship between risk score and prognosis, we randomly divided the patients into two groups (training set and testing set, Supplementary Table S6). For the training set, we plotted a ROC curve and divided BC patients into high- and low-risk groups, and then verified these results in the testing set. We found that patients with high risk scores had poorer prognoses (*p* < 0.05) (Figures 3D–G). Then, we further validated the relationship between risk score and prognosis. The same conclusion was found in the entire set (Figures 3H,I). To validate the predictive ability of the three-mRNA signature, we used the same risk score model to calculate each patient’s risk score associated with OS in the GSE42568 dataset. The 104 patients were ranked by risk score in increasing order (Figure 3J). The mortality rate of patients with high risk scores was higher than that of patients with low risk scores (Figure 3K). The K-M and ROC curves showed that patients with high risk scores had poorer prognosis (*p* = 0.0405) (Figures 3L,M). These results indicate that the risk score could predict the prognosis of patients with BC.

### Risk Score Generated from the Three-mRNA Signature as an Independent Prognostic Indicator

**FIGURE 4:**
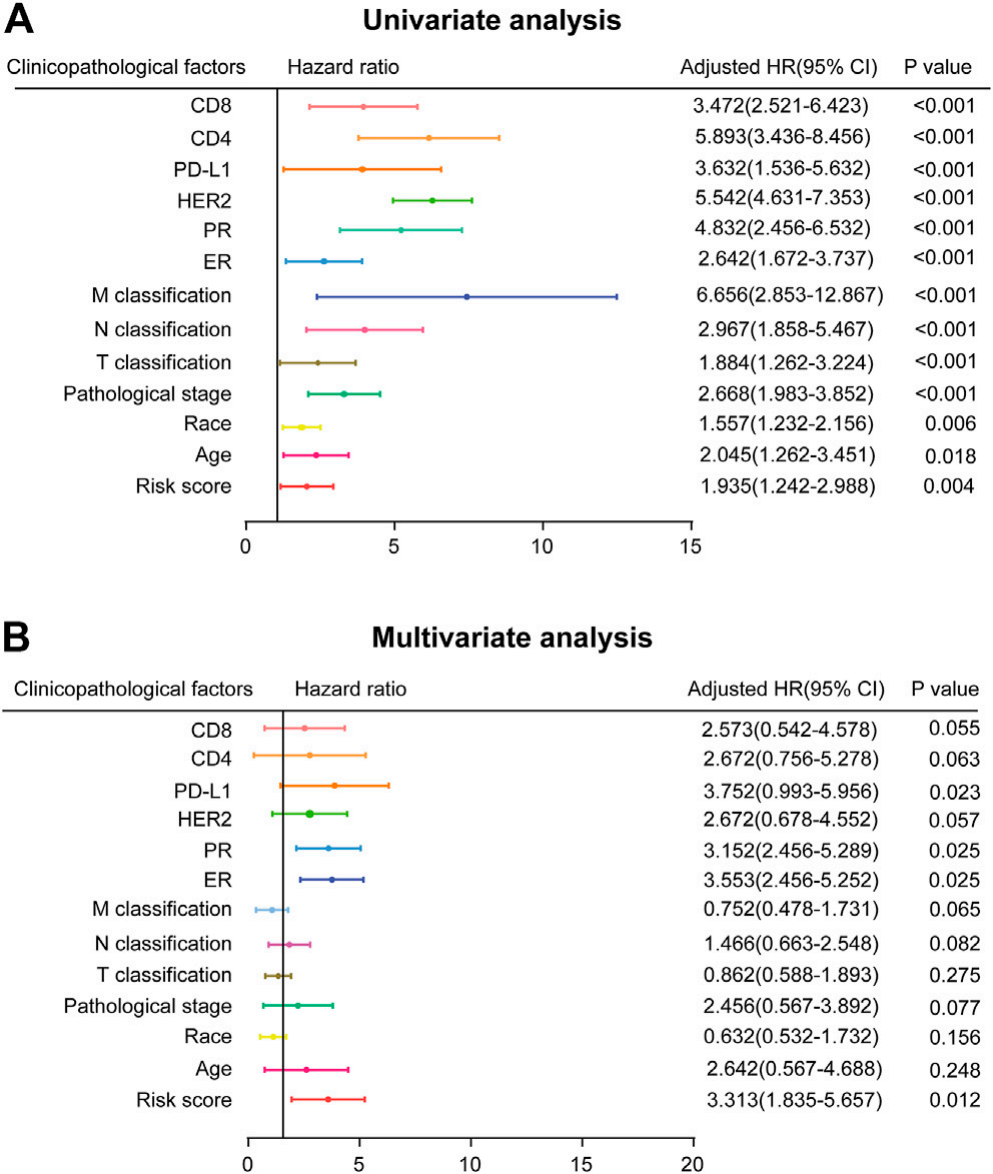
Prognostic value of risk score and clinicopathological parameters in BC patients **(A)** Univariate analysis **(B)** Multivariate analysis (high risk score vs. low risk score; age≥58 vs. <58 years; White vs. Black or African American; I-II vs. III-IV pathological stage; T1-T2 vs. T3-T4 classification; N0-N1 vs. N2-N3 classification; M0 vs. M1 classification; ER, PR, and HER2 negative vs. positive; PD-L1, CD4, and CD8 high expression vs. low expression).

In order to investigate the independence of the prognostic signature, we chose 490 samples with complete clinical information (high vs. low risk score; age≥58 vs. <58 years; White vs. Black or African American; I-II vs. III-IV pathological stage; T1-T2 vs. T3-T4 classification; N0-N1 vs. N2-N3 classification; M0 vs. M1 classification; ER, PR, and HER2 negative vs. positive; PD-L1, CD4, and CD8 high expression vs. low expression) for further analysis by Cox regression (Supplementary Table S7). First, the univariate Cox regression analysis indicated that risk score, age, race, pathological stage, TNM classification, ER, PR, HER2, PD-L1, CD4 and CD8 were prognostic factors for OS of BC patients (Figure 4A). Further multivariate Cox regression analysis confirmed that PD-L1, ER, PR, and risk score were independent prognostic indicators (Figure 4B), showing significant differences not only in the univariate analysis but also in the multivariate analysis (*p* < 0.05). Risk score in particular was remarkably associated with prognosis (*p* = 0.012, HR = 3.313, 95% CI 1.835–5.657).

### K-M Verification of the Survival Prediction of Three-mRNA Signature

**FIGURE 5:**
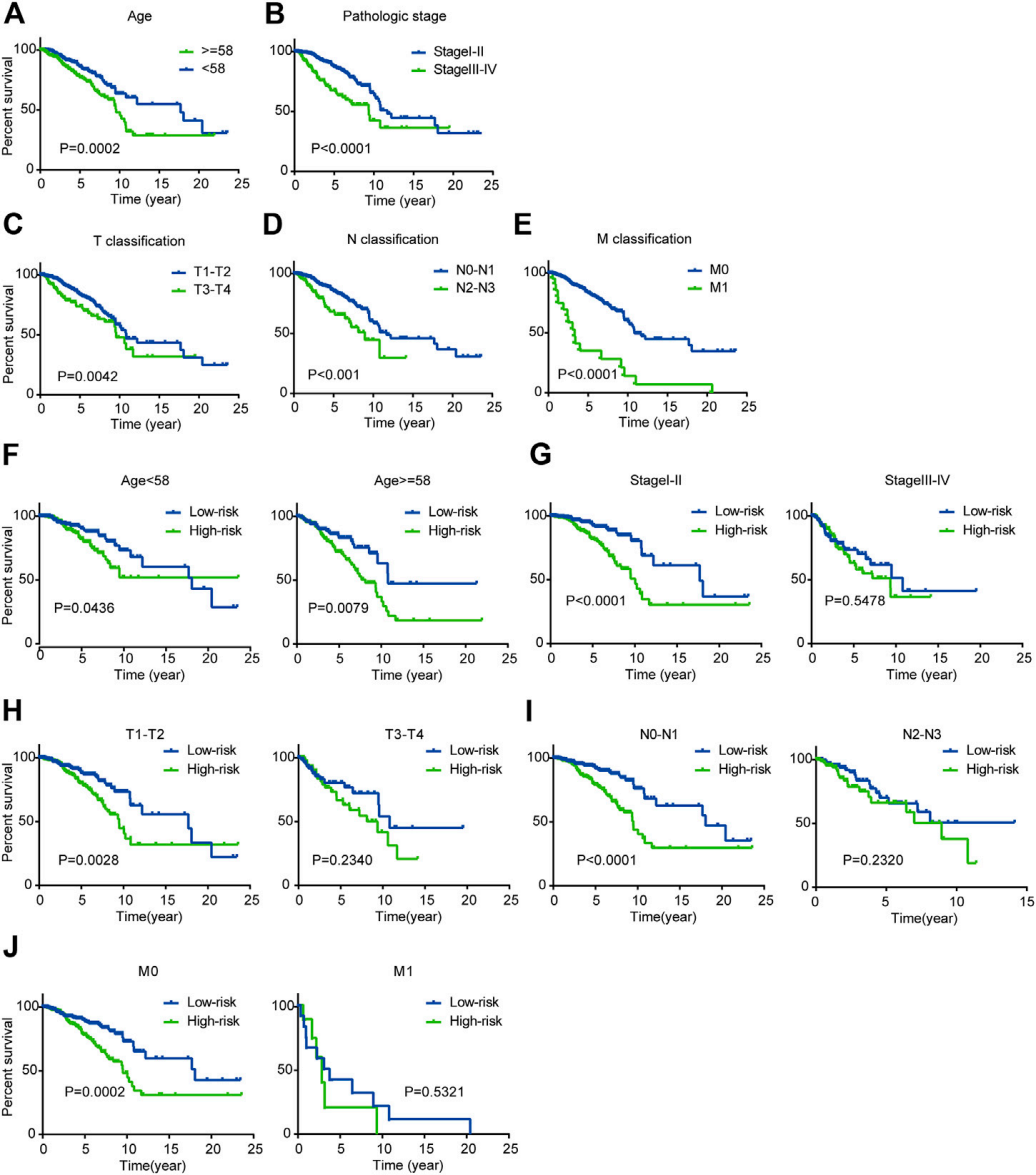
Kaplan-Meier survival analysis for patients with BC in TCGA dataset **(A-E)** Clinical features including age, pathological stage, and TNM classification predict patients’ OS **(F-J)** Kaplan-Meier curves for prognostic value of risk score signature for patients divided by each clinical feature.

As the K-M plot is a univariate method, we performed K-M analysis to verify the reliability of the above prediction and found consistent results. Age, pathological stage, and TNM classification were significantly related to OS of BC patients: BC patients who were older than 58 years, in stage III-IV, and classified as T3-T4, N2-N3, or M1 had poorer prognoses (Figures 5A–E).

Next, we performed stratified analysis for further mining. We grouped patients’ clinical parameters according to their risk scores. As shown by the K-M curves, the three-mRNA signature was a stable prognostic marker for BC independent of age (≥58 or <58 years), in that patients in the high-risk group had poorer prognoses (Figure 5F). However, for patients classified as T1-T2, N0-N1, or M0 and stage I-II, we could use the risk score to predict patient outcomes, with patients in the high-risk subgroup having shorter OS (Figures 5G–J).

### Pathway Analysis to Identify the Mechanism by Which the Gene Signature Regulates Prognosis of BC Patients

**FIGURE 6:**
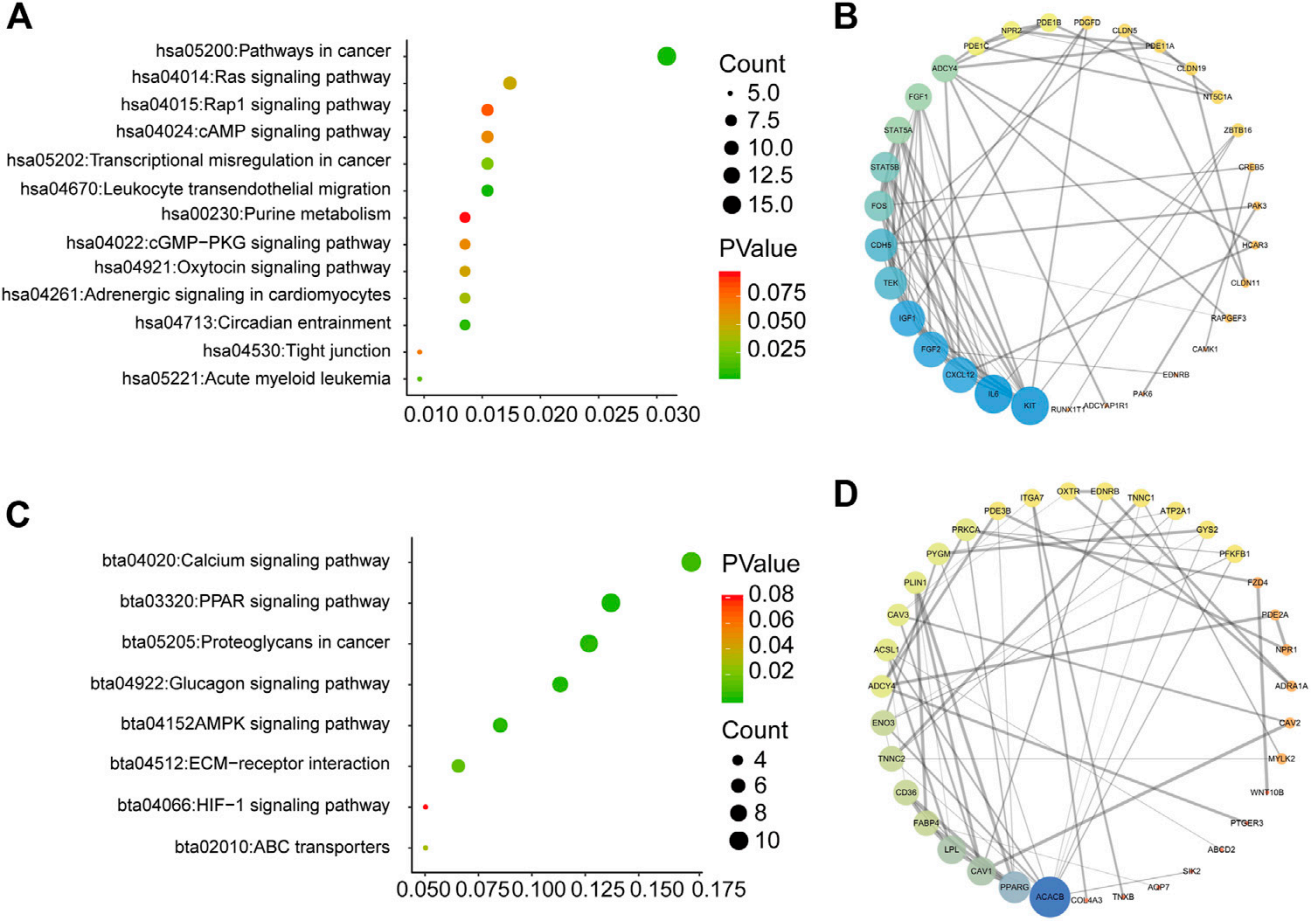
Pathway analysis for genes of low-risk and high-risk BC patients **(A)** KEGG analysis for genes positively correlated with risk score **(B)** PPI network for genes positively correlated with risk score **(C)** KEGG analysis for genes negatively correlated with risk score **(D)** PPI network for genes negatively correlated with risk score.

To explore how the three-mRNA signature regulated the prognosis of BC patients, pathway analysis was carried out on the risk-score-associated genes using the DAVID database. We found that positively related genes were enriched in terms including “pathways in cancer” and “leukocyte transendothelial migration” (Figure 6A). Using these genes, we performed PPI analysis to identify hub genes using the String website and Cytoscape. The results indicated that KIT, IL6, and CXCL12 had crucial roles in the above pathways (Figure 6B). The negatively correlated genes were enriched in “calcium signaling pathway,” “PPAR signaling pathway,” and “proteoglycans in cancer” (Figure 6C), and ACACB, PPARG, CAV1 may be the core of these genes (Figure 6D).

## Discussion

Increasing numbers of mRNAs have been reported as molecular markers of cancer prognosis [26–28]. A panel of biomarkers (CA 125, CYFRA 21-1, HER2 shed antigen, LDH, and CRP) in combination with CEA and CA 15-3 has been shown to increase the sensitivity of early detection of asymptomatic tumor recurrence in BC patients [29]. High expression levels of LDHA and AMPK are associated with shorter OS [30] and AMPK expression is significantly correlated with poor prognosis in BC [31]. However, there have been few attempts to find a robust antigen processing and presentation-related signature to predict the survival outcome of BC patients with respect to tumor immunology. Therefore, it is extremely important to design a new model to predict prognosis in BC so that appropriate treatment measures can be taken.

The key function of the immune system is to identify and confront “foreign aggressors;” however, cancer cells can evade recognition, enabling tumor progression [32–35]. In fact, the establishment of the tumor environment is not the cause of promoting tumor development but the result of failing to recognize tumor antigens effectively [36–39]. Cells with mutant TAP1 (transporter associated with antigen processing 1) have diminished ability to present antigens to CD8^+^ cells, and the expression of TAP1 limits the malignant potential of tumors [40]. Many viruses downregulate or inhibit TAP to evade CTL responses [41]. Thus, antigen processing and presentation is crucial for monitoring the occurrence of tumors.

Among the three genes (HSPA5, PSME2, HLA-F) identified in our study, HSPA5 is a molecular chaperone expressed primarily in the endoplasmic reticulum [42]. The PSME2 gene encodes PA28β, which is a subunit of PA28 [43]. PA28β can activate proteasomes to generate the antigenic peptides presented by MHC class I molecules [44], indicating that it may be related to antigen processing and presentation. Moreover, PA28β can regulate cell invasion of gastric cancer [45]. HLA-F is a non-classical HLA class I antigen; its expression is associated with poor OS and it is a potential prognostic indicator in patients with non-small-cell lung cancer [46]. Ag cross-presentation mediated by HLA-F and MHC-I open conformers cooperate in a MHC-I antigen cross-presentation pathway activated lymphocytes and monocytes, which may significantly promote the regulation of immune system function and defense [47]. HLA-F, as a recently discovered ligand of KIR3DS1, was shown to activate natural killer cells by binding to KIR3DS1 and has been associated with resolution of hepatitis C virus infection [48]. These results indicate that HLA-F is involved in the immune response.

Systemic therapy has become the standard treatment to improve outcomes of patients with operable BC. The purpose of adjuvant systemic therapy is to prolong survival by treating potential micrometastasis [49]. For patients with operable BC, adjuvant therapy is used after surgery and neoadjuvant therapy before surgery. Increasing numbers of clinical trials and studies on adjuvant therapy have shown satisfactory results. For example, neoadjuvant talazoparib has been used in patients with operable BC with a germline BC pathogenic variant [50], cyclin-dependent kinase 4/6 inhibitors are used as a neoadjuvant endocrine therapy for patients with hormone receptor-positive BC [51], and CD73 expression and pathologic stage may influence the effectiveness of neoadjuvant chemotherapy in triple-negative BC [52]. We have developed a three-mRNA signature based on antigen processing and presentation, with a focus on predicting outcomes of all BC patients. An effective combination of this predictive method with neoadjuvant therapy could be of great significance in the diagnosis and treatment of BC.

Based on the relationships with immune cell filtration and the Cox results for independent validation, HLA-F showed the strongest correlation with immune cells. CD4 and CD8 were indicators of poor prognosis, which was surprising. However, it is not difficult to speculate that CD4^+^ T cells, CD8^+^ T cells, and other immune cells could be recruited by tumor cells and exert an immunosuppressive effect on tumor progression. For example, in human BC xenografts in humanized mice, blocking the recruitment of naive CD4^+^ T cells in the tumor by knocking down the expression of PITPNM3, a CCL18 receptor, significantly reduced intragranular regulatory T cells and inhibited tumor progression [16]. Myeloid-derived suppressor cells, immature dendritic cells, and M2 macrophages suppress antitumor immunity and can also promote tumor progression [53, 54]. These findings indicate that immune cells have a dual role in tumor development, which is worthy of further study.

Despite the significant results obtained in the current study, there were inevitably several shortcomings. On the one hand, clinical data of BC patients were downloaded from TCGA. However, the publicly available data contains limited information, so the analysis of clinical pathological parameters in our study was not comprehensive, potentially biasing the results. On the other hand, there have been few previous reports on the roles and signaling mechanisms of these three genes in BC, and no experimental data regarding the identified signature. Therefore, further research is needed to elucidate the inherent correlation between the three-gene signature and the prognosis of BC patients.

In conclusion, we have identified a three-gene signature related to antigen processing and presentation that is associated with OS of BC patients, and shown that this three-gene signature could be an independent factor predicting patient prognosis, with an important role in the early stage of BC. These findings indicate the potential use of a biomarker related to antigen processing and presentation in prognostic assessment in BC, as well as providing theoretical guidance and informing decision‐making regarding BC in clinical practice.

## Data Availability

The original contributions presented in the study are publicly available. The websites of the databases are as follows: Genomic Data Commons Data Portal (https://portal.gdc.cancer.gov/), GEO database (https://www.ncbi.nlm.nih.gov/geo/), GSEA (http://www.broadinstitute.org/gsea/index.jsp), The cBioPortal for Cancer Genomics (http://cbioportal.org), DAVID (https://david.ncifcrf.gov/), ImageGP (http://www.ehbio.com/ImageGP/index.php/Home/Index/index.html), TIMER (https://cistrome.shinyapps.io/timer/), STRING (https://string-db.org/cgi/input?sessionId=b4vwhCQsYnct).
