# Supplementary material for: Development of a Novel Prognostic Signature Based on Antigen Processing and Presentation in Patients with Breast Cancer
Source: Pathol Oncol Res. 2021 Apr 1;27:600727. doi: 10.3389/pore.2021.600727 (PMC8262234; doi:10.3389/pore.2021.600727)
Supplement: Supplementary file 1 [file Table1.DOC]

**Table S1. Clinical pathological parameters of patients with BC**

| **Clinical pathological parameters** | **N** | **%** | **Dead number** |
| --- | --- | --- | --- |
| **Age(years)** |  |  |  |
| >=58 | 551 | 50.55 | 89 |
| <58 | 539 | 49.45 | 60 |
| **Gender** |  |  |  |
| female | 1078 | 98.90 | 136 |
| male | 12 | 1.10 | 1 |
| **T classification** |  |  |  |
| T1-T2 | 912 | 83.67 | 107 |
| T3-T4 | 178 | 16.33 | 40 |
| **N classification** |  |  |  |
| N0-N1 | 880 | 81.71(880/1077) | 103 |
| N2-N3 | 197 | 18.29(880/1077) | 37 |
| **M classification** |  |  |  |
| M0 | 972 | 97.79(972/994) | 120 |
| M1 | 22 | 2.21(22/994) | 17 |
| **Pathological stage** |  |  |  |
| Stage I-II | 804 | 74.93(804/1073) | 81 |
| Stage III-IV | 269 | 25.07(269/1073 | 59 |
| **Menopause stage** |  |  |  |
| peri | 39 | 5.24(39/744) | 1 |
| Post | 705 | 94.76(705/744) | 91 |
| **Cancer status** |  |  |  |
| Tumor free | 878 | 90.33(878/972) | 36 |
| With cancer | 94 | 9.67(94/972) | 60 |
| **HER2 status** |  |  |  |
| negative | 564 | 77.47(564/728) | 57 |
| Positive | 164 | 22.53(164/728) | 23 |

**Table S2. Survival status of BC patients in GSE42568 dataset**

| **Accession** | **Tissue** | **Overall survival time(days)** | **Overall survival event** |
| --- | --- | --- | --- |
| GSM1045208 | breast cancer | 3026 | 0 |
| GSM1045209 | breast cancer | 755 | 1 |
| GSM1045210 | breast cancer | 3014 | 0 |
| GSM1045211 | breast cancer | 1726 | 1 |
| GSM1045212 | breast cancer | 2225 | 0 |
| GSM1045213 | breast cancer | 285 | 1 |
| GSM1045214 | breast cancer | 2138 | 0 |
| GSM1045215 | breast cancer | 2449 | 0 |
| GSM1045216 | breast cancer | 2456 | 1 |
| GSM1045217 | breast cancer | 420 | 1 |
| GSM1045218 | breast cancer | 1110 | 1 |
| GSM1045219 | breast cancer | 488 | 1 |
| GSM1045220 | breast cancer | 2718 | 0 |
| GSM1045221 | breast cancer | 767 | 1 |
| GSM1045222 | breast cancer | 1216 | 1 |
| GSM1045223 | breast cancer | 2462 | 0 |
| GSM1045224 | breast cancer | 1862 | 1 |
| GSM1045225 | breast cancer | 1223 | 1 |
| GSM1045226 | breast cancer | 3019 | 0 |
| GSM1045227 | breast cancer | 155 | 1 |
| GSM1045228 | breast cancer | 947 | 0 |
| GSM1045229 | breast cancer | 552 | 1 |
| GSM1045230 | breast cancer | 2903 | 0 |
| GSM1045231 | breast cancer | 2438 | 0 |
| GSM1045232 | breast cancer | 1627 | 0 |
| GSM1045233 | breast cancer | 1708 | 1 |
| GSM1045234 | breast cancer | 2237 | 0 |
| GSM1045235 | breast cancer | 845 | 1 |
| GSM1045236 | breast cancer | 2957 | 0 |
| GSM1045237 | breast cancer | 913 | 1 |
| GSM1045238 | breast cancer | 138 | 1 |
| GSM1045239 | breast cancer | 1058 | 1 |
| GSM1045240 | breast cancer | 2759 | 0 |
| GSM1045241 | breast cancer | 2764 | 0 |
| GSM1045242 | breast cancer | 1157 | 1 |
| GSM1045243 | breast cancer | 546 | 0 |
| GSM1045244 | breast cancer | 2582 | 0 |
| GSM1045245 | breast cancer | 2460 | 0 |
| GSM1045246 | breast cancer | 1338 | 1 |
| GSM1045247 | breast cancer | 2626 | 0 |
| GSM1045248 | breast cancer | 2948 | 0 |
| GSM1045249 | breast cancer | 2416 | 0 |
| GSM1045250 | breast cancer | 194 | 1 |
| GSM1045251 | breast cancer | 2897 | 0 |
| GSM1045252 | breast cancer | 2899 | 0 |
| GSM1045253 | breast cancer | 2829 | 0 |
| GSM1045254 | breast cancer | 2785 | 0 |
| GSM1045255 | breast cancer | 2740 | 0 |
| GSM1045256 | breast cancer | 1235 | 0 |
| GSM1045257 | breast cancer | 2092 | 1 |
| GSM1045258 | breast cancer | 2535 | 0 |
| GSM1045259 | breast cancer | 2211 | 0 |
| GSM1045260 | breast cancer | 2275 | 1 |
| GSM1045261 | breast cancer | 390 | 1 |
| GSM1045262 | breast cancer | 2184 | 0 |
| GSM1045263 | breast cancer | 919 | 1 |
| GSM1045264 | breast cancer | 917 | 0 |
| GSM1045265 | breast cancer | 2758 | 0 |
| GSM1045266 | breast cancer | 977 | 0 |
| GSM1045267 | breast cancer | 1997 | 0 |
| GSM1045268 | breast cancer | 1854 | 0 |
| GSM1045269 | breast cancer | 1793 | 0 |
| GSM1045270 | breast cancer | 545 | 1 |
| GSM1045271 | breast cancer | 1409 | 1 |
| GSM1045272 | breast cancer | 2943 | 0 |
| GSM1045273 | breast cancer | 1044 | 1 |
| GSM1045274 | breast cancer | 2489 | 0 |
| GSM1045275 | breast cancer | 1332 | 1 |
| GSM1045276 | breast cancer | 1915 | 1 |
| GSM1045277 | breast cancer | 1950 | 0 |
| GSM1045278 | breast cancer | 1080 | 1 |
| GSM1045279 | breast cancer | 2266 | 0 |
| GSM1045280 | breast cancer | 1692 | 0 |
| GSM1045281 | breast cancer | 2302 | 0 |
| GSM1045282 | breast cancer | 365 | 1 |
| GSM1045283 | breast cancer | 2211 | 0 |
| GSM1045284 | breast cancer | 2344 | 0 |
| GSM1045285 | breast cancer | 1864 | 0 |
| GSM1045286 | breast cancer | 2484 | 0 |
| GSM1045287 | breast cancer | 526 | 1 |
| GSM1045288 | breast cancer | 2955 | 0 |
| GSM1045289 | breast cancer | 2650 | 0 |
| GSM1045290 | breast cancer | 2384 | 0 |
| GSM1045291 | breast cancer | 2689 | 0 |
| GSM1045292 | breast cancer | 2641 | 0 |
| GSM1045293 | breast cancer | 423 | 0 |
| GSM1045294 | breast cancer | 1682 | 0 |
| GSM1045295 | breast cancer | 2052 | 0 |
| GSM1045296 | breast cancer | 475 | 0 |
| GSM1045297 | breast cancer | 2243 | 0 |
| GSM1045298 | breast cancer | 2197 | 0 |
| GSM1045299 | breast cancer | 2576 | 0 |
| GSM1045300 | breast cancer | 2556 | 0 |
| GSM1045301 | breast cancer | 2225 | 0 |
| GSM1045302 | breast cancer | 2022 | 1 |
| GSM1045303 | breast cancer | 2713 | 0 |
| GSM1045304 | breast cancer | 2790 | 0 |
| GSM1045305 | breast cancer | 2622 | 0 |
| GSM1045306 | breast cancer | 2909 | 0 |
| GSM1045307 | breast cancer | 2952 | 0 |
| GSM1045308 | breast cancer | 2989 | 0 |
| GSM1045309 | breast cancer | 2105 | 0 |
| GSM1045310 | breast cancer | 927 | 1 |
| GSM1045311 | breast cancer | 2962 | 0 |
| **Total** | **104** | **Dead numer** | **35** |

**Table S3. 32 genes derived from GSEA**

| PROBE | RANK IN GENE LIST | RANK METRIC SCORE | RUNNING ES | CORE ENRICHMENT |
| --- | --- | --- | --- | --- |
| TAP2 | 27172 | -0.285942435 | -0.5197894 | Yes |
| KIR2DL4 | 27201 | -0.287092566 | -0.5069914 | Yes |
| HLA-DQA2 | 27466 | -0.295971543 | -0.5006817 | Yes |
| HLA-DRA | 27543 | -0.29837051 | -0.488754 | Yes |
| HLA-DPB1 | 27607 | -0.30049175 | -0.47634512 | Yes |
| HLA-DRB1 | 28090 | -0.318761826 | -0.47533688 | Yes |
| CD74 | 28190 | -0.322931856 | -0.46291757 | Yes |
| CTSB | 28238 | -0.324287713 | -0.44891152 | Yes |
| HLA-F | 28625 | -0.340348393 | -0.44406873 | Yes |
| HLA-B | 29508 | -0.382358998 | -0.4517548 | Yes |
| HLA-DQA1 | 29576 | -0.385694295 | -0.4354216 | Yes |
| HLA-DQB1 | 29628 | -0.388168067 | -0.41850257 | Yes |
| CANX | 29850 | -0.398520172 | -0.4060697 | Yes |
| HLA-A | 29858 | -0.398800761 | -0.38735816 | Yes |
| NFYA | 29876 | -0.399658859 | -0.36889866 | Yes |
| HSP90AA1 | 30173 | -0.415102899 | -0.35787502 | Yes |
| LTA | 30232 | -0.418513298 | -0.3397216 | Yes |
| HSPA8 | 30524 | -0.435503721 | -0.32758388 | Yes |
| HLA-C | 31249 | -0.481499255 | -0.32594156 | Yes |
| IFI30 | 31309 | -0.485711932 | -0.30462992 | Yes |
| PSME3 | 31575 | -0.50528425 | -0.28842106 | Yes |
| TAP1 | 31858 | -0.525934935 | -0.2717304 | Yes |
| HSP90AB1 | 32075 | -0.543852985 | -0.2522575 | Yes |
| RFXANK | 32305 | -0.566741765 | -0.2320795 | Yes |
| PSME1 | 33086 | -0.657368779 | -0.22373463 | Yes |
| HSPA4 | 33173 | -0.673629761 | -0.1942999 | Yes |
| HSPA5 | 33407 | -0.709454954 | -0.16746965 | Yes |
| PSME2 | 33690 | -0.773955822 | -0.13901448 | Yes |
| TAPBP | 33718 | -0.778763473 | -0.10286553 | Yes |
| PDIA3 | 33781 | -0.796523511 | -0.066898875 | Yes |
| CALR | 33826 | -0.808801174 | -0.029822845 | Yes |
| RFX5 | 33992 | -0.87253511 | 0.006733732 | Yes |

**Table S4. 14 genes derived from univariate Cox regression analysis**

| **gene** | **HR** | **z** | **pvalue** |
| --- | --- | --- | --- |
| HSPA5 | 0.60746126 | -4.742734999 | 2.11E-06 |
| PSME2 | 0.572229493 | -4.504589092 | 6.65E-06 |
| RFXANK | 0.653835254 | -3.930990956 | 8.46E-05 |
| TAPBP | 0.609035606 | -3.674346418 | 0.000238459 |
| PDIA3 | 0.669766786 | -3.582868514 | 0.000339842 |
| CALR | 0.629694673 | -3.375666156 | 0.000736372 |
| HSP90AB1 | 0.689755204 | -3.246798157 | 0.001167111 |
| HLA-DRB1 | 0.802348728 | -3.072957217 | 0.002119489 |
| PSME3 | 0.827217215 | -2.702544481 | 0.006881098 |
| HLA-DQA1 | 0.858754673 | -2.576552364 | 0.009979109 |
| HLA-B | 0.866022471 | -2.406084629 | 0.016124529 |
| HLA-F | 0.841586122 | -2.186577741 | 0.028773364 |
| KIR2DL4 | 0.907831489 | -2.12887681 | 0.033264454 |
| HSP90AA1 | 0.9092005 | -2.054209205 | 0.039955448 |

**Table S5. 3 genes derived from multivariate Cox regression analysis**

| **mRNA** | **Ensemble ID** | **Location** | **Β (Cox)** | **HR** | **P** |
| --- | --- | --- | --- | --- | --- |
| HSPA5 | ENSG00000044574 | chr9:125,234,853-125,241,330 | -0.439 | 0.661 | 2.11E-06 |
| PSME2 | ENSG00000100911 | chr14:24,143,362-24,147,570 | -0.414 | 0.644 | 6.65E-06 |
| HLA-F | ENSG00000204642 | chr6:29,722,775-29,738,528 | 0.213 | 1.238 | 0.0288 |

**P value, HR** were assessed using univariate Cox regression analysis; **HR:** hazard ratio.

**Table S6. Survival status of BC patients in training set and** **testing set**

|  | **N** | **%** | **Dead number** |
| --- | --- | --- | --- |
| training set | 530 | 66.7% | 72 |
| testing set | 265 | 33.3% | 39 |

**Table S7. Clinical pathological parameters of 490 samples for Cox analysis**

| **Clinical pathological parameters** | **N** | **%** | **Dead number** |
| --- | --- | --- | --- |
| **Risk score** |  |  |  |
| high | 268 | 54.69 | 46 |
| low | 222 | 45.31 | 21 |
| **Age(years)** |  |  |  |
| >=58 | 237 | 48.37 | 39 |
| <58 | 253 | 51.63 | 28 |
| **Race** |  |  |  |
| White | 358 | 73.06 | 42 |
| Black or African American | 132 | 26.94 | 25 |
| **T classification** |  |  |  |
| T1-T2 | 415 | 84.69 | 17 |
| T3-T4 | 75 | 15.31 | 50 |
| **N classification** |  |  |  |
| N0-N1 | 398 | 81.22 | 24 |
| N2-N3 | 92 | 18.78 | 43 |
| **M classification** |  |  |  |
| M0 | 381 | 77.76 | 26 |
| M1 | 109 | 22.24 | 41 |
| **Pathological stage** |  |  |  |
| Stage I-II | 375 | 76.53 | 12 |
| Stage III-IV | 115 | 23.47 | 55 |
| **HER2 status** |  |  |  |
| negative | 421 | 85.92 | 44 |
| Positive | 69 | 14.08 | 23 |
| **ER status** |  |  |  |
| negative | 123 | 25.10 | 28 |
| Positive | 367 | 74.90 | 39 |
| **PR status** |  |  |  |
| negative | 147 | 30.00 | 20 |
| Positive | 343 | 70.00 | 47 |
| **PDL-1 status** |  |  |  |
| high | 284 | 57.96 | 30 |
| low | 206 | 42.04 | 37 |
| **CD4 status** |  |  |  |
| high | 241 | 49.18 | 41 |
| low | 249 | 50.82 | 26 |
| **CD8 status** |  |  |  |
| high | 283 | 57.76 | 46 |
| low | 207 | 42.24 | 21 |
